# Efficient gene deletion of Integrin alpha 4 in primary mouse CD4 T cells using CRISPR RNA pair-mediated fragmentation

**DOI:** 10.3389/fimmu.2024.1445341

**Published:** 2024-12-10

**Authors:** Taeuk Wi, Yurim Choi, Jungsun Kim, Youn Soo Choi, Matthew E. Pipkin, Jinyong Choi

**Affiliations:** ^1^ Department of Microbiology, College of Medicine, The Catholic University of Korea, Seoul, Republic of Korea; ^2^ Department of Medical Sciences, Graduate School of The Catholic University of Korea, Seoul, Republic of Korea; ^3^ Department of Biomedical Sciences, Department of Medicine, Seoul National University College of Medicine, Seoul, Republic of Korea; ^4^ Transplantation Research Institute, Seoul National University Hospital, Seoul, Republic of Korea; ^5^ Department of Immunology and Microbiology, The Herbert Wertheim UF Scripps Institute for Biomedical Innovation and Technology, Jupiter, FL, United States

**Keywords:** integrin α4, CRISPR-Cas9 gene editing, T_FH_, T_H_1, viral infection

## Abstract

The functional specialization of CD4 T lymphocytes into various subtypes, including T_H_1 and T_FH_ cells, is crucial for effective immune responses. T_FH_ cells facilitate B cell differentiation within germinal centers, while T_H_1 cells are vital for cell-mediated immunity against intracellular pathogens. Integrin α4, a cell surface adhesion molecule, plays significant roles in cell migration and co-stimulatory signaling. In this study, we investigated the role of Integrin α4 in regulating T_FH_ and T_H_1 cell populations during acute viral infection using CRISPR-Cas9 gene editing. To effectively delete the *Itga4* in primary mouse CD4 T cells, we selected various combinations of crRNAs and generated ribonucleoprotein complexes with fluorochrome-conjugated tracrRNAs and Cas9 proteins. These crRNA pairs enhanced gene deletion by generating deletions in the gene. By analyzing the effects of *Itga4* deficiency on T_FH_ and T_H_1 cell differentiation during acute LCMV infection, we found that optimized crRNA pairs significantly increased the T_H_1 cell population. Our results highlight the importance of selecting and combining appropriate crRNAs for effective CRISPR-Cas9 gene editing in primary CD4 T cells. Additionally, our study demonstrates the role of Integrin α4 in regulating the differentiation of CD4 T cells, suggesting the potential molecular mechanisms driving T cell subset differentiation through integrin targeting.

## Introduction

The adaptive immune response relies heavily on the functional specialization of CD4 T lymphocytes, which can differentiate into various subtypes, including T helper type 1 (T_H_1), T_H_2, T_H_17, regulatory T (T_REG_), and follicular helper T (T_FH_) cells (1). Among these, T_FH_ cells play a crucial role in the formation of germinal centers (GCs) and the differentiation of B cells, which are essential for humoral immunity (2). T_FH_ cells are characterized by the expression of C-X-C chemokine receptor 5 (CXCR5), which facilitates their migration towards the B cell follicles, and B-cell lymphoma 6 (Bcl6), a transcription factor (TF) that upregulates T_FH_-associated genes, including cytokines and costimulatory molecules, while repressing genes that inhibit T_FH_ cell development (2–4). Conversely, T_H_1 cells are essential for cell-mediated immunity and are involved in the defense against intracellular pathogens, such as viruses and certain bacteria. Additionally, they play a role in anti-tumor immunity (5, 6). Maintaining a balance between T_H_1 and T_FH_ cell populations is critical for an effective immune response, ensuring both adequate antibody production and effective cellular immunity.

Integrins, which are cell surface adhesion molecules, are critical for cell-cell interactions, migration, and co-stimulatory signaling (7–9). Integrin α4, in particular, forms heterodimers with Integrin β1 (VLA-4) or Integrin β7 (LPAM-1), and these complexes play significant roles in transendothelial migration and immune cell localization. VLA-4 and LPAM-1 interact with vascular cell adhesion molecule 1 (VCAM-1) and mucosal addressin cell adhesion molecule 1 (MAdCAM-1), respectively, facilitating adhesion and migration signals essential for immune cell trafficking (10–12). Furthermore, Integrin α4β1 (VLA-4) is known to promote T_H_1 responses by upregulating interferon (IFN)-γ expression and suppressing interleukin (IL)-4, a key cytokine of T_H_2 cells (13).

CRISPR/Cas9 gene editing has emerged as a powerful tool for investigating gene function and regulation in various biological contexts, including immune cell differentiation and function (14). This technology utilizes guide RNAs to direct the Cas9 nuclease to specific genomic loci, where it induces double-strand breaks. These breaks are typically repaired by the non-homologous end joining (NHEJ) pathway, resulting in insertions or deletions (InDels) that can disrupt gene function (15, 16). The efficiency of gene disruption using CRISPR/Cas9 can be enhanced by employing multiple CRISPR RNAs (crRNAs) targeting different sites within the same gene, thereby increasing the likelihood of generating large deletions and achieving complete gene disruption (17–21).

In the current study, we investigated the role of Integrin α4/CD49d, encoded by *Itga4*, in regulating the balance between T_FH_ and T_H_1 cell populations during acute viral infection. We used CRISPR/Cas9 gene editing to delete *Itga4* in primary mouse CD4 T cells. We adopted an advanced crRNA/Cas9 ribonucleoprotein (crRNP) complex-mediated gene editing strategy to analyze *Itga4* disruption efficiency and nucleotide deletion patterns based on the length of base pairs between target sites using two crRNAs. We then examined the effects of *Itga4* deficiency on T_FH_ and T_H_1 cell differentiation during acute virus infection. Our study provides insights into the molecular mechanisms driving T cell subset differentiation, highlighting the potential role of Integrin α4 in regulating T_H_1 cell proliferation or differentiation during acute viral infection. Furthermore, we present efficient crRNP-mediated gene deletion methods using *Itga4* as an example gene in mouse primary CD4 T cells for their use in *in vivo* experiments.

## Materials and methods

### Mice

C57BL/6 mice (6 weeks old) were acquired from Orient Bio in Korea. CD45.1^+^ SMARTA mice were generously provided by Yoon Soo Choi at Seoul National University College of Medicine. Specific-pathogen-free male or female donor mice (6-11 weeks old) were used for experiments. All procedures involving animals were conducted in compliance with the protocol approved by the Institutional Animal Care and Use Committee of the College of Medicine at the Catholic University of Korea (CUMC-2023-0268).

### CD4 T cell isolation

Spleens from WT C57BL/6 or CD45.1^+^ SMARTA mice were collected. CD4 T cells were isolated through a negative selection process according to the manufacturer’s protocol (EasySep™ Mouse CD4 T cell Isolation kit, STEMCELL Technologies). To isolate naive CD4 T cells, biotinylated anti-CD44 (IM7, Biolegend) and anti-CD25 (PC61, Biolegend) were added to the Isolation Cocktail of the Mouse CD4 T cell Isolation kit. After isolation, cells were counted, and the proportion of CD4 T cells was assessed by flow cytometry. The purity of the isolated CD4 T cells exceeded 95%. The isolated WT or SMARTA CD4 T cells were used for *in vitro* CD4 T cell culture or *in vivo* adoptive transfer experiments.

### *In vitro* CD4 T cell culture

The tissue culture plate (24-well) was coated with 330 µl of 8 µg/ml anti-CD3ϵ (145-2C11; BioXCell) and 8 ug/mL anti-CD28 (37.51; BioXCell) in PBS (Corning). Isolated CD4 T cells were plated at 0.5 × 10^6^ cells/well in R10 media (RPMI 1640 with 10% Fetal Bovine Serum (Hyclone), 100 U/ml Penicillin, 100 µg/ml Streptomycin, GlutaMAX™), supplemented with 50 µM 2-Mercaptoethanol (2-ME, Gibco), Non-Essential Amino Acids (NEAA, Gibco) and 2 ng/mL recombinant human IL-7 (rhIL-7, Peprotech), and cultured for 2 days.

### CRISPR/Cas9-mediated gene deletion of mouse CD4 T cells

Various crRNAs targeting *Itga4* were selected based on their CHOP-CHOP ranking and the distances between crRNAs (https://chopchop.cbu.uib.no) (**Supplementary Table 1**). A crRNA targeting *Cd8a* was used as a control. crRNAs, ATTO-550-conjugated trans-activating CRISPR RNA (tracrRNA), and S.p Cas9 Nuclease V3 were purchased from Integrated DNA Technologies (IDT). crRNA and tracrRNA were duplexed by heating at 95 °C for 5 minutes and incubated at room temperature for 1 hour in the dark. crRNP complexes were generated by mixing crRNA-tracrRNA duplexes (40~240 pmol range) and Cas9 protein (26.6~80 pmol range) for 10 minutes at room temperature. *In vitro* stimulated CD4 T cells were harvested, resuspended in 20 µl of primary cell nucleofector solution (P4 Primary Cell 4D-Nucleofector™, Lonza), and mixed with the prepared crRNPs complexes. The crRNP-cell mixtures were then transferred to a 16-well Nucleocuvette stirp and electroporated using the CM137 (for *in vitro* activated CD4 T cells) or DS137 (for naive CD4 T cell) protocol with the 4D-Nucleofector™ (Lonza). The transfected cells were cultured in R10 media supplemented with 50 µM 2-ME, NEAA, and 10 ng/mL recombinant human IL-2 (rhIL-2, Peprotech) without TCR stimulation. Two days later, the transfected CD4 T cells were sorted by FACS Aria fusion (BD Bioscience). The sorted cells were further used for validation of the efficiency of gene delivery by flow cytometry and gene editing by Inference of CRISPR Edits (ICE) analysis or used for adoptive transfer experiments. Gene deletion efficiency, InDel proportion, and InDel contributions by each crRNA from triplicate wells were measured initially, with minimal variations observed between triplicate wells (**Supplementary Figures 1A–D**). We conducted two to four independent experiments for further analysis.

### DNA sequencing and Inference of CRISPR Edits (ICE) analysis

Genomic DNA of the sorted CD4 T cells was prepared using QIAamp DNA Mini Kit according to the manufacturer’s protocol (Qiagen). PCR was performed for crRNA target site amplification of crRNAs using Q5 polymerase (NEB Biolabs) following the manufacturer’s protocol. Sanger sequencing was performed by Macrogen or Bionics (Korea). ICE analysis was conducted using Synthego online platform (https://ice.synthego.com). The InDel contributions were calculated based on the sum of InDel percentages generated by the crRNA, as provided in the ICE analysis report (**Supplementary Figure 1A**). For example, the InDel contributions of cr*Itga4* B (g1), cr*Itga4* C (g2), and the deletions in the gene resulting from cr*Itga4* B and *crItga4* C (referred to as “fragment” from paired crRNAs) were 20 (11 + 4 + 3 + 1+1), 4 (2 + 1 + 1), and 76 (36 + 34 + 2 + 1+1 + 1+1), respectively, when using the combination of cr*Itga4* B and cr*Itga4* C (**Supplementary Figure 1A**).

### Adoptive cell transfer and virus infection

1 × 10^3^ freshly isolated or 3 × 10^4^ sorted crRNP^+^ CD45.1^+^ SMARTA CD4 T cells were adoptively transferred into C57BL/6 recipient mice via intravenous injection into the retroorbital sinus. Recipient mice were then injected intraperitoneally with 2 × 10^5^ pfu of LCMV_Arm_ in plain DMEM either one day later (for freshly isolated CD4 T cells) or 3 to 4 days later (for crRNP^+^ CD4 T cells) following the adoptive transfer.

### *In vitro* T_H_1 differentiation and cell proliferation assays

Naive CD4 T cells were electroporated with crRNP and cultured for 2 days in R10 media supplemented with 50 µM 2-ME, NEAA, and 2 ng/mL rhIL-7. For T_H_1 differentiation, 1.0 × 10^6^ cells/well were cultured in 24-well plates pre-coated anti-CD3 and soluble anti-CD28 antibodies, along with 10 ng/mL recombinant murine IL-12 p70 (rmIL-12, Peprotech) and 10 µg/mL anti-IL-4 (11B11; BioXcell) for 3 days, as previously described (22). For the T_H_0 control, cells were cultured without rmIL-12 and anti-IL-4 antibody. After 3 days, cells were split at a 1:2 ratio and cultured in fresh rhIL-2 medium for an additional 2 days. For cytokine analysis, cells were stimulated with 50 ng/ml PMA and 1 µM ionomycin for 5 hours. To assess cell proliferation, cells cultured for 3 days under T_H_0 and T_H_1 differentiation conditions were labeled with CellTrace Violet (CTV; CellTrace™ Violet Cell Proliferation Kit, Invitrogen), and then cultured in fresh rhIL-2 medium for an additional 2 days. Cytokine expression and CTV levels were measured by flow cytometry.

### Flow cytometry

Surface staining for flow cytometry was performed using monoclonal antibodies against CD8 (53-6.7; APC-Cy7), B220 (RA3-6B2; APC-Cy7), CD4 (RM-4; BV510 or PE-Cy7), CD45.1 (A20; BV605 or APC-Cy7), TCRVα2 (B20.1; FITC), Integrin α4 (R1-2; APC or PE), Integrin β1 (HMb1-1; APC-eF780), Integrin β7 (FIB504; PE.CF594), CD44 (IM7; PE-Cy7), SLAM (TC15-12F12.2; PerCP-Cy5.5 or APC), PD-1 (J43; PE), biotin-conjugated CXCR5 (L138D7) and BV421, PE-Cy7 or BV650-conjugated streptavidin. The antibody cocktail was diluted in FACS buffer (PBS with 0.5% Bovine Serum Albumin). Staining was performed in FACS buffer at 4°C for 30 minutes. For CXCR5 staining, biotinylated anti-CXCR5 was used, followed by a 30-minute incubation at 4°C with BV421, PE-Cy7 or BV650-conjugated streptavidin. Live/dead cell staining was carried out using Fixable Viability Dye eFlour™780 (eBioscience). Intracellular cytokine staining was conducted with monoclonal antibody IFN-γ (XMG1.2; AF700) using the Fixation/Permeabilization kit (BD Biosciences). Intranuclear staining for the transcription factor was performed with a monoclonal antibody with Bcl6 (K112-91; APC), T-bet (4B10; PerCP-Cy5.5), GATA3 (16E10A23; APC), FOXP3 (FJK-16s; PerCP-Cy5.5), Ki-67 (B56; PE-TexasRed) utilizing the Foxp3/Transcription Factor Staining Buffer kit (eBioscience). Stained cells were analyzed using a FACS Canto II or LSRFortessa (BD Biosciences), and data analyses were conducted with FlowJo software v.10.10.0 (FlowJo). The gating strategy used to identify T_FH_ and T_H_1 populations is shown in **Supplementary Figure 2**.

### RNA sequencing data analysis

Transcripts per Million (TPM) values of *Bcl6* and *Itga4* for WT T_H_1 and WT T_FH_ cells (GSE140187) were obtained from RNA-seq data previously published (24).

### Analysis of BCL6 chromatin immunoprecipitation with sequencing

The UCSC tracks and peaks at the *ITGA4* locus from BCL6 ChIP–seq data of human tonsillar GC T_FH_ cells (GSE59933) were retrieved from earlier publications (23, 24). Peaks were annotated according to the RefSeq database. Peaks located within ±2 kb of the transcription start site were classified as promoter peaks, those within ±2 kb of the transcription end site were defined as 3′ end peaks, and peaks located more than 2 kb away from genes were categorized as intergenic, following methods described in previous work (24).

## Results

### The expression of Integrin α4 is downregulated in T_FH_ cells

**Figure 1:**
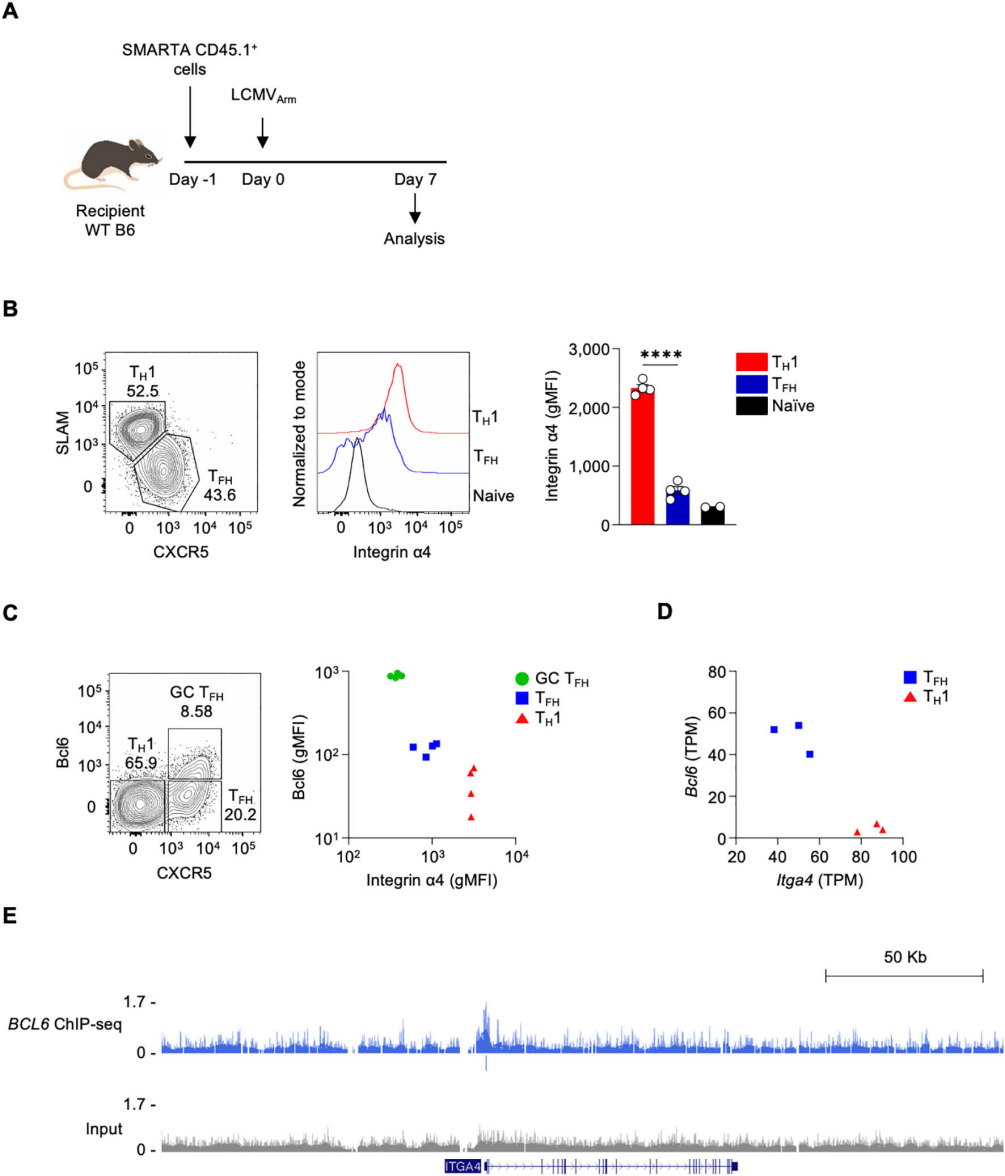
The expression of integrin α4 is downregulated in T_FH_ cells **(A)** Schematic diagram of the adoptive transfer of SMARTA CD4 T cells in LCMV_Arm_ infection model. Isolated splenic SMARTA CD45.1^+^ CD4 T cells were adoptively transferred into C57BL/6 mice, followed by LCMV_Arm_ infection, and analyzed on day 7 post-infection. **(B)** T_FH_ and T_H_1 populations were analyzed by flow cytometry. Integrin α4 expression levels in the T_FH_ and T_H_1 populations are shown. **(C)** Bcl6 and Integrin α4 expression levels in the GC T_FH_, T_FH_, and T_H_1 populations are shown. A representative of two independent experiments is shown, and each dot represents one mouse (n=4). **(D)** Gene expression levels of *Bcl6* and *Itga4* in T_FH_ and T_H_1 cells from RNA-seq data of LCMV_Arm_-infected mice. **(E)** Genome browser tracks display a BCL6 ChIP–Seq peak at *ITGA4* locus, with peak annotations shown the track. Results are presented as mean ± SEM and were analyzed using an unpaired two-tailed Student’s t-test. ****p < 0.0001.

We previously showed that T_FH_ cells exhibited lower *Itga4* RNA expression compared to T_H_1 cells during acute lymphocytic choriomeningitis virus (LCMV) infection (24). To investigate whether the protein expression of Integrin α4 correlates with the RNA expression changes in different CD4 T cell populations, including T_FH_ and T_H_1 cells, we utilized adoptive transfer of antigen-specific CD4 T cells in an acute virus infection mouse model. The mice were adoptively transferred with I-A^b^-restricted LCMV glycoprotein (gp)-specific SMARTA CD4 T cells, followed by infection with LCMV Armstrong (LCMV_Arm_) (**Figure 1A**). Consistent with observations from the previous study (24), LCMV_Arm_ infection generated CXCR^lo^ SLAM^hi^ T_H_1 and CXCR5^hi^ SLAM^lo^ T_FH_ populations in the spleen (**Figure 1B**) on day 7 post-infection. The expression level of Integrin α4 was measured in each population. Both T_H_1 and T_FH_ populations expressed higher amounts of Integrin α4 protein than naive CD4 T cells. Consistent with the RNA expression data, T_FH_ cells showed significantly lower expression of Integrin α4 than T_H_1 cells (**Figure 1B**).

As *Itga4* was observed as a potential direct Bcl6 target gene (24), we analyzed the correlation between the expression of Bcl6 and Integrin α4 in GC T_FH_, T_FH_, and T_H_1 populations. GC T_FH_, which are the highest Bcl6-expressing cells, express the lowest amount of Integrin α4; in contrast, T_H_1, which are the lowest Bcl6-expressing cells, express the highest amount of Integrin α4 (**Figure 1C**). Additionally, RNA-seq analysis revealed a negative correlation between *Bcl6* and *Itga4* mRNA expression (**Figure 1D**) (24). To assess whether Bcl6 binds to the regulatory region of *Itga4*, we analyzed BCL6 ChIP-seq data from human tonsillar GC T_FH_ cells (23, 24). Indeed, BCL6 binding was detected at the *ITGA4* promoter region, indicating that Bcl6 may directly repress *Itga4* expression (**Figure 1E**). These results indicate differential expression of Integrin α4 across distinct T cell subsets following LCMV_Arm_ infection, with the lowest expression observed in GC T_FH_ cells, demonstrating a negative correlation between Bcl6 and Integrin α4 expression. This differential expression pattern suggests a potential role for Integrin α4 in the differentiation, functional specialization, or migratory behavior of these CD4 T cell subsets during the immune response to LCMV_Arm_ infection.

### Combinations of proximal crRNA pairs efficiently delete Integrin α4 in CD4 T cells

**Figure 2:**
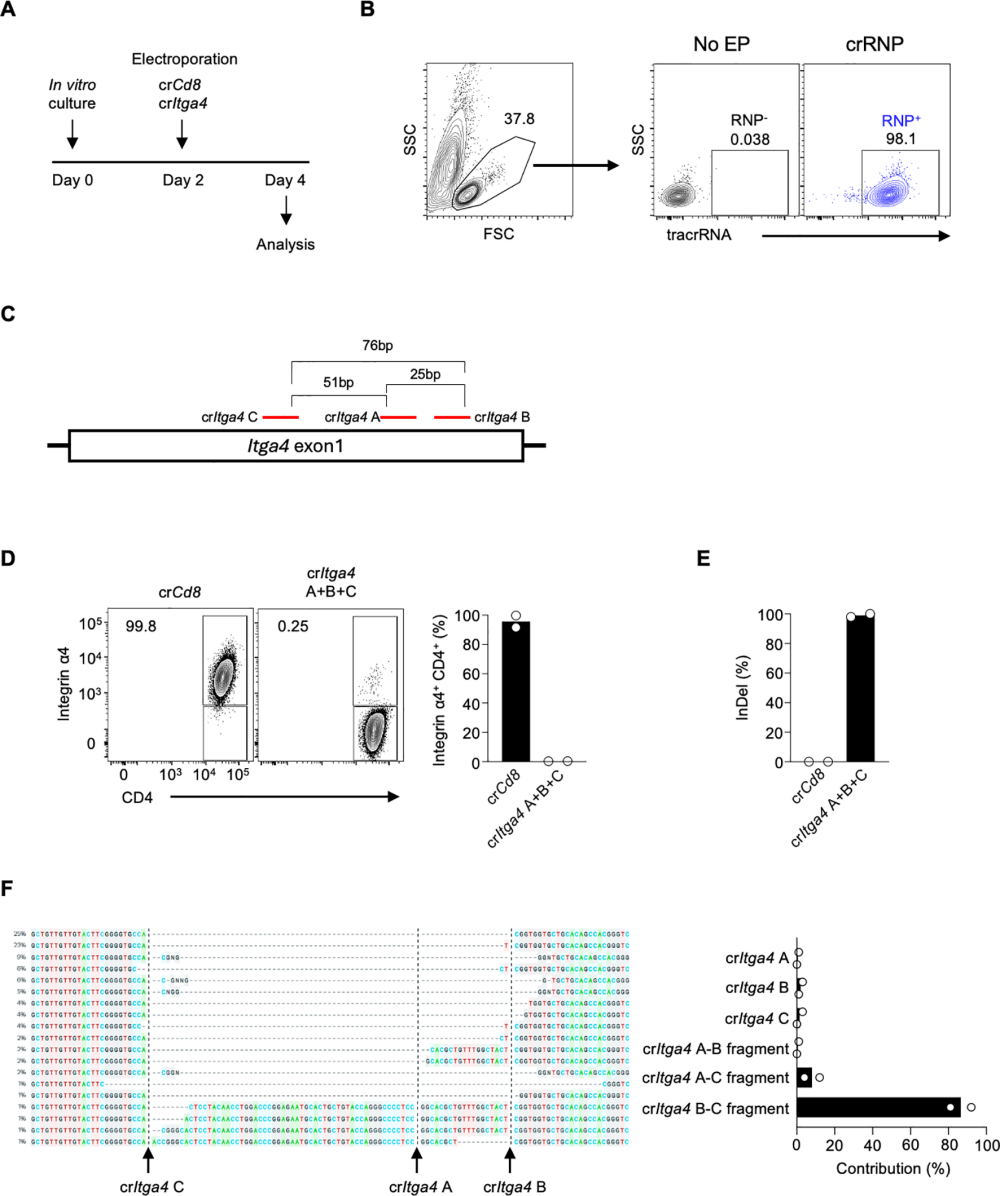
Combining multiple crRNAs efficiently deletes integrin α4 in CD4 T cells **(A)** Schematic diagram of the *in vitro* culture and electroporation protocol. Mouse splenic CD4 T cells were isolated and stimulated with anti-CD3/CD28 and IL-7 for two days. On day 2, cells were electroporated with either control crRNP (cr*Cd8*) or crRNP targeting *Itga4* (cr*Itga4*), followed by analysis on day 4. **(B)** Electroporation efficiency of crRNP delivery was measured by flow cytometry. **(C)** Diagram of crRNA target sites in *Itga4* exon 1. **(D)** Flow cytometry analysis of Integrin α4 expression in CD4 T cells. A mixture cr*Itga4* A+B+C was generated with 80 pmol each of cr*Itga4* A, B, and C (total 240 pmol) and used for electroporation **(E)** ICE analysis of InDel percentage. The bar graph shows the percentage of InDels in the cr*Cd8* and cr*Itga4* groups. **(F)** Sequencing analysis of InDel contributions by each crRNA. The alignment shows the contribution of each crRNA and its combinations to the observed gene editing. The contributions represent the inferred sequences present in the crRNP population and their relative proportions. Actual cut sites are represented by black vertical dotted lines. Arrows mark the double-strand breakage sites by each crRNA. Data shown are representative of two independent experiments.

To investigate the role of Integrin α4 in different CD4 T cell populations in LCMV infection, we employed acute gene deletion using CRISPR-Cas9 gene editing (17, 24). Mouse splenic CD4 T cells were isolated and stimulated with anti-CD3/CD28 and IL-7 for two days *in vitro*, followed by electroporation with the crRNP complex, and the delivery efficiency and deletion efficiency were analyzed two days later (**Figure 2A**). The tracrRNA^+^ provides electroporation efficiency as the crRNP complex contains fluorescence-conjugated tracrRNA. The tracrRNA^+^ population in the crRNP electroporated group was higher than 98%, while it was not observed in no electroporation control (No EP), indicating that the electroporation of the crRNP complex was successful (**Figure 2B**).

Previous studies have shown that using combinations of dual or triple crRNAs enhances gene deletion efficiency in human primary T cells by generating insertion and deletion (InDel)-mediated fragment deletions (17, 19, 20). Initially, we designed three different crRNAs targeting *Itga4* (cr*Itga4* A, B, and C; **Supplementary Table 1**). These crRNAs all target exon 1 of the *Itga4* gene, with distances between crRNAs being 25 bp (cr*Itga4* A – cr*Itga4* B), 51 bp (cr*Itga4* A – cr*Itga4* C), and 76 bp (cr*Itga4* B – cr*Itga4* C) (**Figure 2C**). To maximize gene deletion efficiency, we used a mixture of all three crRNAs (cr*Itga4* A+B+C). The Integrin α4 positive population in the control cr*Cd8* group was higher than 95%, but in cr*Itga4* A+B+C group, it decreased to less than 1%, indicating that a mixture of crRNAs targeting *Itga4* is highly efficient in disruption of *Itga4* gene expression (**Figure 2D**).

We further analyzed DNA editing efficacy using Inference of CRISPR Edits (ICE) analysis, which can locate the site of InDel and calculate the proportion of InDel by sequencing PCR products that include crRNA target sites. The InDel percentage of cr*Itga4* A+B+C was higher than 90%, indicating cr*Itga4* A+B+C was highly efficient in generating DNA editing (**Figure 2E**). Interestingly, although a mixture of three different crRNAs was delivered into the cells, each crRNA targeting *Itga4* contributed differently to the gene editing. Most of the InDel was contributed by cr*Itga4* B and cr*Itga4* C, which can generate around a 76-bp fragment deletion (**Figure 2F**). Although cr*Itga4* A was ranked as the top crRNA by CHOP CHOP, the contributions of a cr*Itga4* A and fragments containing cr*Itga4* A (cr*Itga4* A–B or cr*Itga4* A–C fragment) were less than 5% (**Figure 2F**). It is possible that the effect of cr*Itga4* A could not be measured because cr*Itga4* A targets a site in the middle between cr*Itga4* B and cr*Itga4* C. These findings suggest that using a pair of crRNAs may be more efficient than using a combination of three crRNAs if the pair is sufficient to effectively delete the target gene.

**Figure 3:**
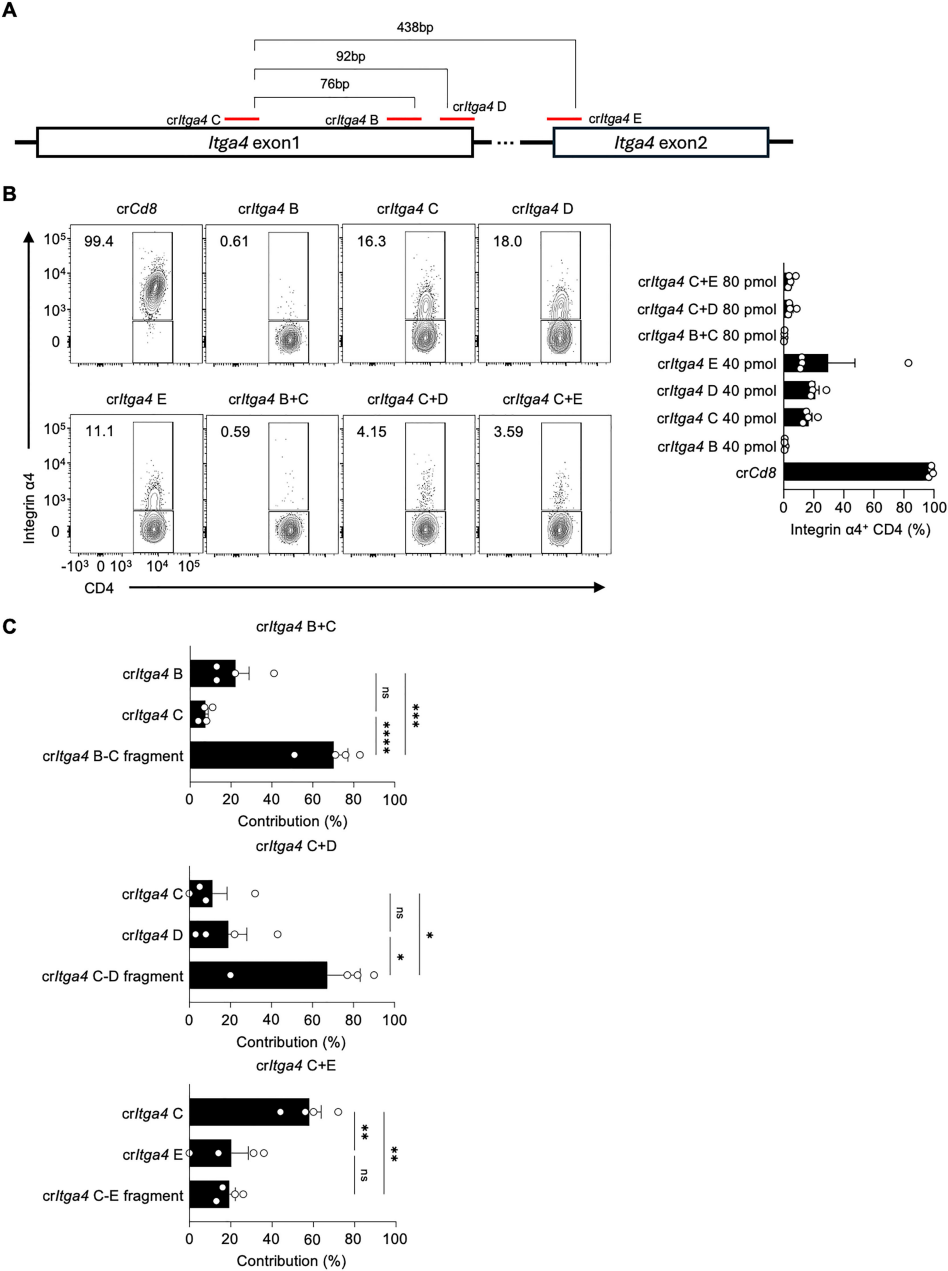
Combinations of proximal crRNA pairs efficiently delete integrin α4 in CD4 T cells **(A)** Diagram of crRNA target sites in *Itga4* exons 1 and 2. **(B)** Flow cytometry analysis of Integrin α4 expression in CD4 T cells after electroporation with single or paired crRNAs. Single cr*Itga4* was used at 40 pmol, and a mixture cr*Itga4* pair was generated with 40 pmol of each cr*Itga4* (total 80 pmol). The bar graph quantifies the percentage of Integrin α4^+^ CD4 T cells for each condition. **(C)** Sequencing analysis of InDel contributions of crRNAs to the overall InDel profile of crRNA pairs. Data shown are representative of four independent experiments. Results are presented as mean ± SEM and were analyzed using one-way ANOVA. ns, non-significant; *p < 0.05; **p < 0.01; ***p < 0.001; ****p < 0.0001.

Given that crRNA-induced fragmentation and their locations are important for enhancing gene deletion efficiency, we further tested additional crRNAs targeting the *Itga4* gene, including cr*Itga4* E targeting exon 2 to generate fragments of varying lengths (**Figure 3A**; **Supplementary Table 1**). We also investigated if a lower amount of crRNP could suffice for effective gene deletion. Mouse splenic CD4 T cells were electroporated with either a single (40 pmol) or a mixture of dual crRNAs (total 80 pmol), which is one-sixth or one-third of the original 240 pmol total crRNA, respectively (**Figure 3B**). The use of single cr*Itga4* B completely abolished Integrin α4 expression, whereas the efficiencies of cr*Itga*4 C, D, or E were similar, leaving 11~18% of Integrin α4^+^ populations (**Figure 3B**). Notably, combining two crRNAs, cr*Itga4* C+D or cr*Itga4* C+E, significantly enhanced disruption efficiency, as evidenced by less than 5% of the Integrin α4^+^ population remaining (**Figure 3B**).

We examined the InDel contributions of crRNAs in the mixture groups in more detail. Intriguingly, although cr*Itga4* C+E, with a distance of 438 bp between the two crRNAs, generated almost complete deletion of Integrin α4 expression, the InDel contribution was primarily from cr*Itga4* C rather than from the cr*Itga4* C–E fragment (**Figure 3C**). This suggests that the fragmentation effect may be influenced by the distance between the two crRNAs. Among the crRNA mixtures, cr*Itga4* C+D produced the most synergistic effect on gene disruption and fragment generation (**Figures 3B, C**). These results demonstrate the importance of crRNA selection and combination for efficient gene deletion, providing insights into optimizing CRISPR-Cas9 strategies for targeted gene editing.

### Deficiency of *Itga4* in CD4 T cells increases the accumulation of T_H_1 cells in the spleen during acute viral infection

**Figure 4:**
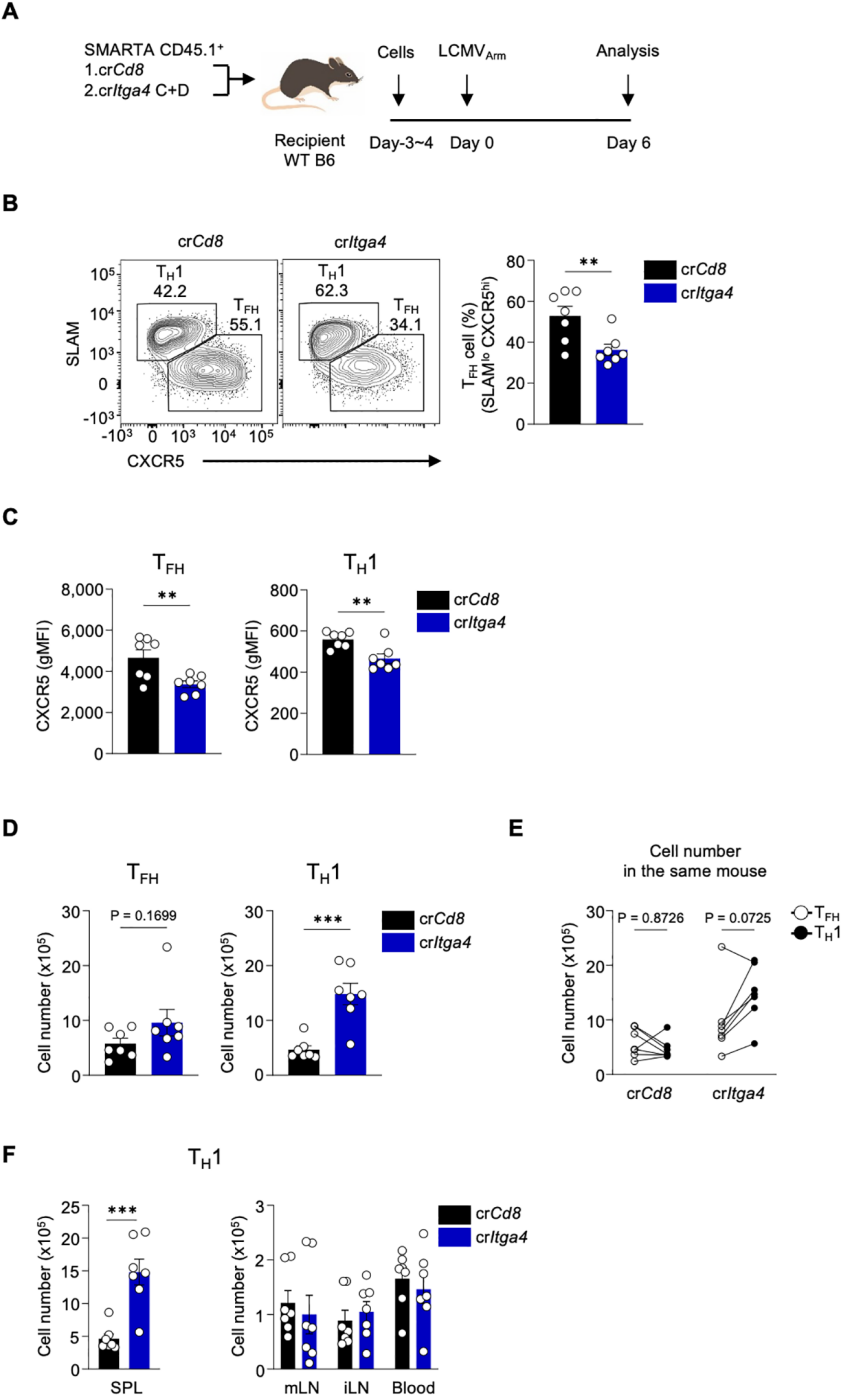
Deficiency of *Itga4* in CD4 T cells increases the accumulation of T_H_1 cells in the spleen during acute viral infection **(A)** SMARTA CD4 T cells were transfected with either cr*Cd8* or cr*Itga4* C+D, adoptively transferred into C57BL/6 recipient mice, followed by LCMV_Arm_ infection, and analyzed on day 6 post-infection. A mixture of cr*Itga4* C+D was generated with 40 pmol of each cr*Itga4* (total 80 pmol). **(B)** T_FH_ and T_H_1 populations were analyzed by flow cytometry. **(C)** Expression levels of CXCR5 were calculated in T_FH_ and T_H_1 cells. **(D)** Cell numbers of T_FH_ and T_H_1 populations were calculated. **(E)** Comparative cell numbers of T_FH_ and T_H_1 populations within the same mouse were calculated. The graph shows the cell numbers of T_FH_ and T_H_1 populations in individual mice receiving either control cr*Cd8* or cr*Itga4* C+D transfected SMARTA CD4 T cells. A representative of two independent experiments is shown, and each dot represents one mouse (n=7). Results are presented as mean ± SEM and were analyzed by using unpaired **(B–D, F)** or paired **(E)** two-tailed Student’s t-test. **p < 0.01; ***p < 0.001.

To assess the role of *Itga4* deficiency in CD4 T cells during acute viral infection, we adoptively transferred SMARTA CD4 T cells, either transfected with cr*Itga4* C+D or control cr*Cd8*, into WT B6 recipient mice. Following LCMV_Arm_ infection, we analyzed the cells on day 6 post-infection (**Figure 4A**). Most cells from the cr*Itga*4 group were Integrin α4-negative during viral infection (**Supplementary Figure 3A**). Interestingly, the lack of Integrin α4 also led to a decrease in expression of its heterodimeric partners, Integrin β1/CD29 and Integrin β7 (**Supplementary Figure 3B**). This outcome likely results from the process by which integrins are translated into proteins, paired with their partner integrin subunits to form a heterodimer before being expressed on the cell surface (8).

We initially hypothesized that the deficiency of *Itga4* might increase the T_FH_ population since its expression level is downregulated in T_FH_ cells (**Figure 1**). In stark contrast, a significant decrease in the proportion of CXCR5^hi^ SLAM^lo^ T_FH_ cells was observed in the cr*Itga4* group compared to the cr*Cd8* control group, whereas the proportion of CXCR5^lo^ SLAM^hi^ T_H_1 cells was increased (**Figure 4B**). Furthermore, CXCR5 expression was significantly reduced in both T_FH_ and T_H_1 cells in the cr*Itga4* group, while PD-1 levels remained unchanged (**Figure 4C**; **Supplementary Figure 4A**). Notably, Bcl6 expression was also reduced in T_FH_ (**Supplementary Figure 4B**), whereas the levels of T-bet (the lineage defining TF of T_H_1), GATA-3 (T_H_2), and Foxp3 (T_REG_) remained unchanged in the T_H_1 population in LCMV-infected mice (**Supplementary Figures 4B, C**). These findings suggest that Integrin α4 signaling may play a role in inducing Bcl6 and CXCR5, indicating that *Itga4* deficiency may alter T_FH_ phenotypic characteristics.

To further investigate changes in the proportions of T_FH_ and T_H_1 cells, we quantified the cell numbers in each population. There was no significant difference in the total number of T_FH_ cells between the two groups. However, the cr*Itga4* group showed an apparent increase in the number of T_H_1 cells (**Figure 4D**). Consistently, there were more T_H_1 cells than T_FH_ cells in the cr*Itga4* group, whereas no significant difference was found between these subsets in the cr*Cd8* group (**Figure 4E**). To better understand this increase T_H_1 population, we tested whether the accumulation was due to enhanced proliferation following Integrin α4 disruption. We evaluated Ki-67 levels in T_H_1 cells *in vivo*, but found no significant differences between the cr*Cd8* and cr*Itga4* groups in LCMV-infected mice (**Supplementary Figure 4D**). We further examined whether *Itga4* disruption affected T_H_1 proliferation *in vitro*, as Ki-67 expression in *in vivo* T_H_1 cells might reflect the steady-state level. *Itga4* was ablated, followed by *in vitro* T_H_1 differentiation. Interestingly, *Itga4* disruption significantly increased the proliferation of T_H_1 cells while reducing the proliferation of T_H_0 cells (**Supplementary Figure 4E**). This suggests that Integrin α4 may regulate CD4 T cell proliferation differently, depending on TCR and IL-12 cytokine signaling pathways. We also investigated whether *Itga4* disruption affected T_H_1 differentiation. In contrast to the findings for T_H_1 cell proliferation, the frequency of IFN-γ-producing T_H_1 cells decreased in the cr*Itga4* group (**Supplementary Figure 4F**). Therefore, the accumulation of T_H_1 cells in the cr*Itga4* group may result from the combined effects of both proliferation and differentiation.

Since Integrin α4 is known to regulate CD4 T cell trafficking and homing, we examined the distribution of T_H_1 cells across various lymphoid tissues, including the spleen, mesenteric lymph nodes (mLNs), inguinal lymph nodes (iLNs), and blood. No significant differences in T_H_1 populations were observed between the cr*Cd8* and cr*Itga4* groups in mLN, iLN, and blood. However, the T_H_1 population in the spleen of the cr*Itga4* group was significantly higher than in the cr*Cd8* group (**Figure 4F**), suggesting that T_H_1 cells may preferentially accumulate in the spleen over other lymphoid organs. In conclusion, *Itga4* deficiency in CD4 T cells may skew the immune response towards a T_H_1 phenotype by specifically controlling T_H_1 cell accumulation in the spleen and regulating CD4 T cell proliferation during acute LCMV infection. This highlights a potential role for Integrin α4 in balancing T_FH_ and T_H_1 cell populations during antiviral immune responses.

## Discussion

In the current study, we demonstrate the potentially important role of Integrin α4 in regulating the balance between T_FH_ and T_H_1 cell populations during acute viral infection. The downregulation of Integrin α4 in T_FH_ cells, compared to its upregulation in T_H_1 cells, aligns with our previous analysis of its expression at the RNA level (24). This differential expression suggests that Integrin α4 may be involved in the functional specialization and migratory behavior of these T cell subsets and also serve as a useful marker for T_FH_ and T_H_1 cells in viral infection or vaccination.

Integrins are known to be involved in cell-cell interaction, localization, migration, and co-stimulatory functions (7–9). For instance, VLA-4 (Integrin α4β1) plays a role in TCR-mediated CD4 T cell activation by facilitating CD3-dependent downstream signaling (9). Moreover, stimulation of VLA-4 with monoclonal antibodies against Integrin α4 and Integrin β1 induces T cell proliferation (25). While a high-affinity form of the integrin LFA-1 is known to regulate the development and maintenance of T_FH_ cells (26), the role of VLA-4 in the regulation of T_FH_ and T_H_1 subsets has remained unclear. The significant reduction in Integrin α4 expression in T_FH_ cells suggests a possible mechanism that helps these cells to be retained in specific microenvironments, such as germinal centers, whereas higher Integrin α4 expression in T_H_1 cells aids their distribution to inflammatory sites. Indeed, the conjugation of VLA-4 is known to mediate the polarization of T_H_1 cells (13). Interestingly, the deficiency of *Itga4* in CD4 T cells led to a notable shift in T cell populations, with an increased accumulation of T_H_1 cells in the spleen, contrary to our initial hypothesis that *Itga4* deficiency might favor T_FH_ cell development. This indicates that Integrin α4 plays a potential role in maintaining the balance between these subsets by regulating T_H_1 accumulation. It is possible that Integrin α4 may influence activation signaling for the differentiation, proliferation, or migration of T_H_1 cells. *Itga4* deficiency showed increased *in vitro* T_H_1 cell proliferation, which may ultimately affect T_H_1 accumulation. Future studies are needed to further explore how Integrin α4 regulates spleen-specific accumulation, proliferation, or migration of T_H_1 cells. Moreover, the altered expression of CXCR5 in T_FH_ cells from the *Itga4*-deficient group suggests that signaling pathways downstream of VLA-4 may impact the optimal localization to the B cell follicle and stable maintenance of T_FH_ cell features despite its low expression in T_FH_ cells. Further research is warranted to investigate the localization and functional impact of *Itga4*-deficient T_FH_ and T_H_1 populations.

The utilization of CRISPR-Cas9 gene editing allowed us to effectively disrupt *Itga4* expression in CD4 T cells. Combinations of multiple crRNA mixtures have been tried in human primary T cells and mouse CD8 T cells with varied gene deletion efficiency (17, 19–21). Our results also showed that combinations of proximal crRNA pairs achieved high deletion efficiency, as evidenced by the substantial reduction of Integrin α4 expression. Interestingly, the analysis of InDel contributions revealed that certain crRNA pairs within the combination were more effective in deletions in the gene. For cr*Itga4* C, D, and E, the gene deletion efficacy of single use of these crRNA was similar. The distance between the crRNAs may be an unignorable factor for efficient fragmentation and gene deletion. Indeed, crRNA pairs with 32 ~ 51 bp interval targeting *TET2*, *DOT1L*, and *PRDM1* in human primary T cells generated highly effective gene deletion, while a mixture of three different crRNAs targeting *Itgav*/CD51 with distance longer than 10 ~ 75 kb in mouse CD8 T cells left 25 ~ 75% of CD51 positive populations (19, 21). Our research suggests that the distance between crRNA pairs, between approximately 70 and 100 bp and shorter than 400 bp, might be effective for deletions in the gene. Furthermore, reducing the amounts of crRNA through optimized crRNA paring and Cas9 protein usage could enhance cost-effectiveness. These findings emphasize the importance of strategic crRNA selection to optimize gene editing outcomes, suggesting a robust approach for gene deletion in primary T cells.

Our study elucidates an important role of Integrin α4 in regulating T_FH_ and T_H_1 cell dynamics during acute viral infection. The use of CRISPR-Cas9 gene editing to disrupt *Itga4* expression in CD4 T cells provided valuable insights into the molecular mechanisms driving T cell subset differentiation. Future studies should explore the detailed mechanism of Integrin α4 on T_H_1 regulation, broader implications of integrin-mediated T cell regulation, and investigate potential therapeutic applications for modulating immune responses through integrin targeting.

## Data Availability

The original contributions presented in the study are included in the article/**Supplementary Material**. The data presented in the study are deposited in the Sequence Read Archive (SRA) repository, BioProject accession number PRJNA1183801. Further inquiries can be directed to the corresponding author.
