# Supplementary material for: Efficient gene deletion of Integrin alpha 4 in primary mouse CD4 T cells using CRISPR RNA pair-mediated fragmentation
Source: Front Immunol. 2024 Dec 10;15:1445341. doi: 10.3389/fimmu.2024.1445341 (PMC11666438; doi:10.3389/fimmu.2024.1445341)
Supplement: Supplementary file 1 [file DataSheet1.pdf]

Supplementary Material

Supplementary Tables

crRNA	CHOP CHOP Rank	Position	Sequence
Itga4 A	1	Exon 1	5’-TAGCCAAACAGCGTGCCGGAGGG-3’
Itga4 B	5	Exon 1	5’-CACGCTGTTTGGCTACTCGGTGG-3’
Itga4 C	8	Exon 1	5’-TGTACTTCGGGGTGCCAACCGGG-3’
Itga4 D	19	Exon 1	5’-CTCACCAGCGCTTCGACCCGTGG-3’
Itga4 E	20	Exon 2	5’-CTTGCTTTAGGCTCATCGTGGGG-3’
Cd8a	55	Exon 1	5’-GCAGGTTCAGCGACAGAAAGCGG-3’

Supplementary Table 1. crRNA sequences targeting *Itga4*
crRNAs targeting *Itga4* are listed along with their CHOP CHOP ranking, positions within the target gene, and the corresponding sequences. The cr*Cd8a* crRNA is included as a control. The protospacer adjacent motif (PAM) sequences are underlined. The rankings indicate the predicted efficiency and specificity of each crRNA as determined by the CHOP CHOP algorithm.

Supplementary Figures

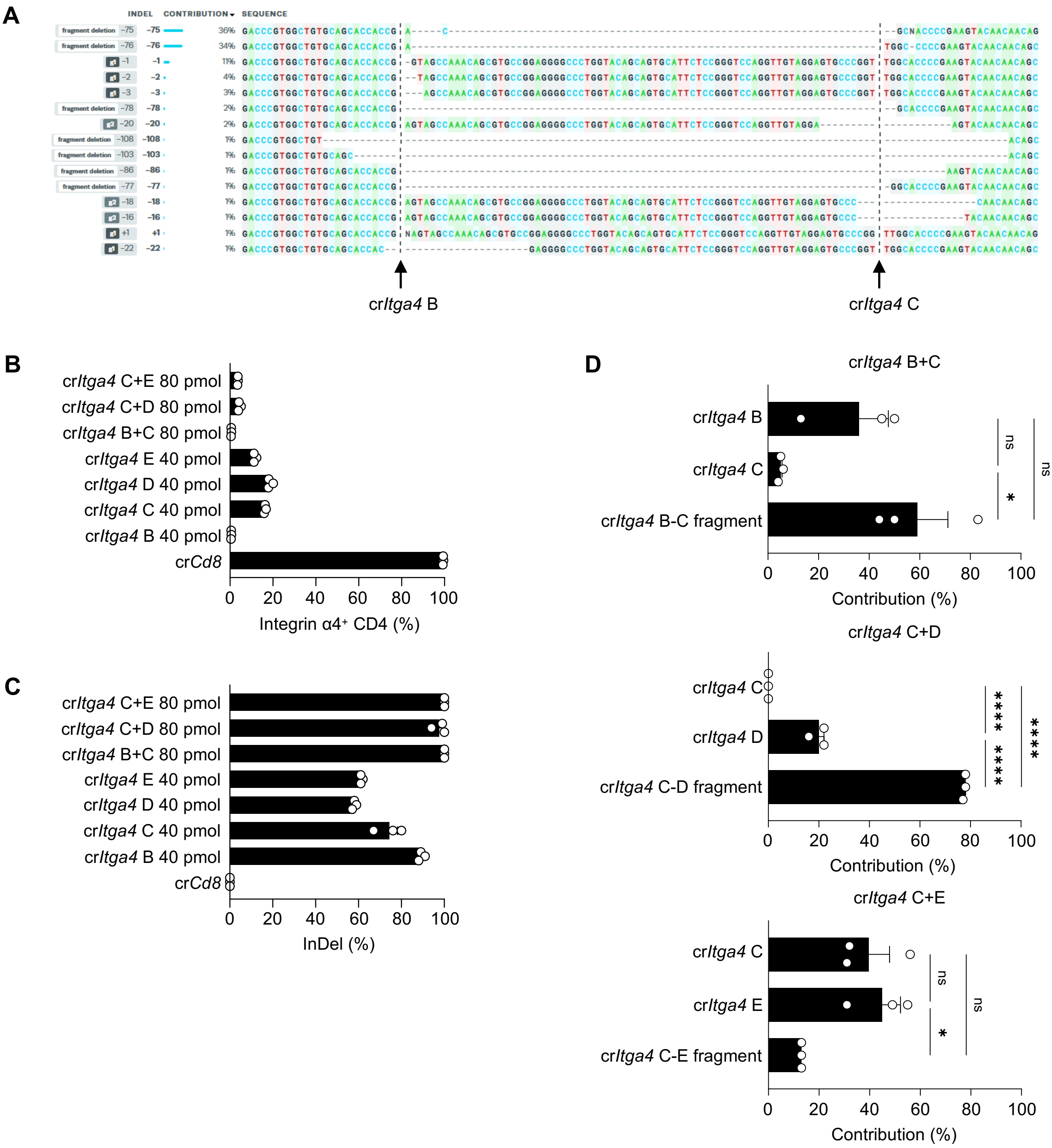

Supplementary Figure 1. Gene deletion efficiency of cr*Itga4*s in CD4 T cells (A) The alignment shows the contribution of each crRNA and its combinations to the observed gene editing. Actual cut sites are represented by black vertical dotted lines (http://ice.synthego.com). Arrows mark the double-strand breakage sites by each crRNA. (B,C) Gene deletion efficiency of single and paired cr*Itga4* in mouse CD4 T cells is evaluated through flow cytometry and sequencing analysis between triplicate wells. (D) The graph represents the contribution of each cr*Itga4* generated by the proximal cr*Itga4* pair to the total InDel. Results are presented as mean ± SEM and were analyzed using an one-way ANOVA. ns, non-significant; *$p < 0.05$; ****$p < 0.0001$.

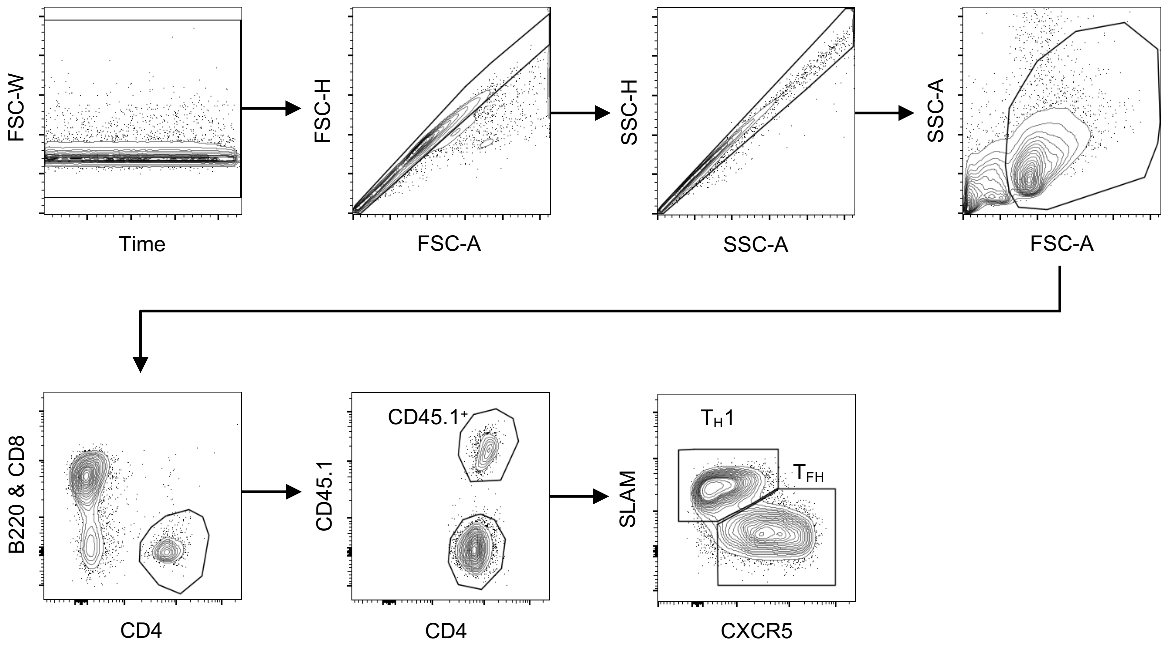

Supplementary Figure 2. Gating strategy for identifying T_H1 and T_{FH} cell in splenocytes

The gating strategy used to identify the T_H1 and T_{FH} populations in the spleen of $LCMV_{Arm}$-infected mice. Adoptively transferred SMARTA $CD45.1^+$ CD4 T cells express CD45.1, which allows them to be distinguished from endogenous $CD45.2^+$ CD4 T cells. $SLAM^{hi}$ $CXCR5^{lo}$ cells and $SLAM^{lo}$ $CXCR5^{hi}$ cells are defined as T_H1 ands T_{FH} cells, respectively.

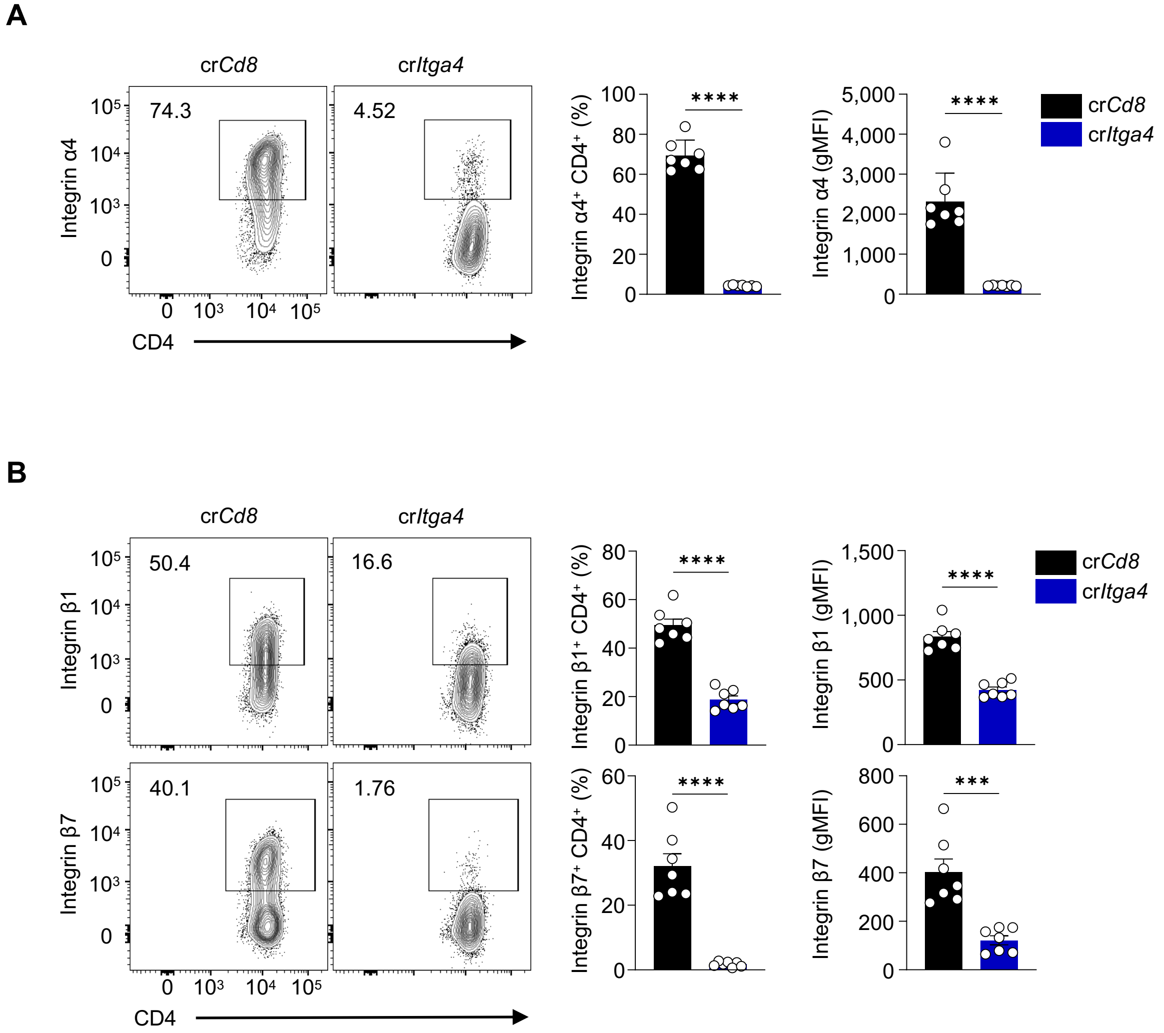

Supplementary Figure 3. Validation of *Itga4* disruption and its effects on Integrin β1 and β7 expression (A, B) Flow cytometry analysis of Integrin α4, Integrin β1, and Integrin β7 expression in SMARTA CD4 T cells during acute viral infection. A representative of two independent experiments is shown, and each dot represents one mouse (n=7). Results are presented as mean ± SEM and were analyzed by using unpaired two-tailed Student's t-test. ***p < 0.001; ****p < 0.0001.

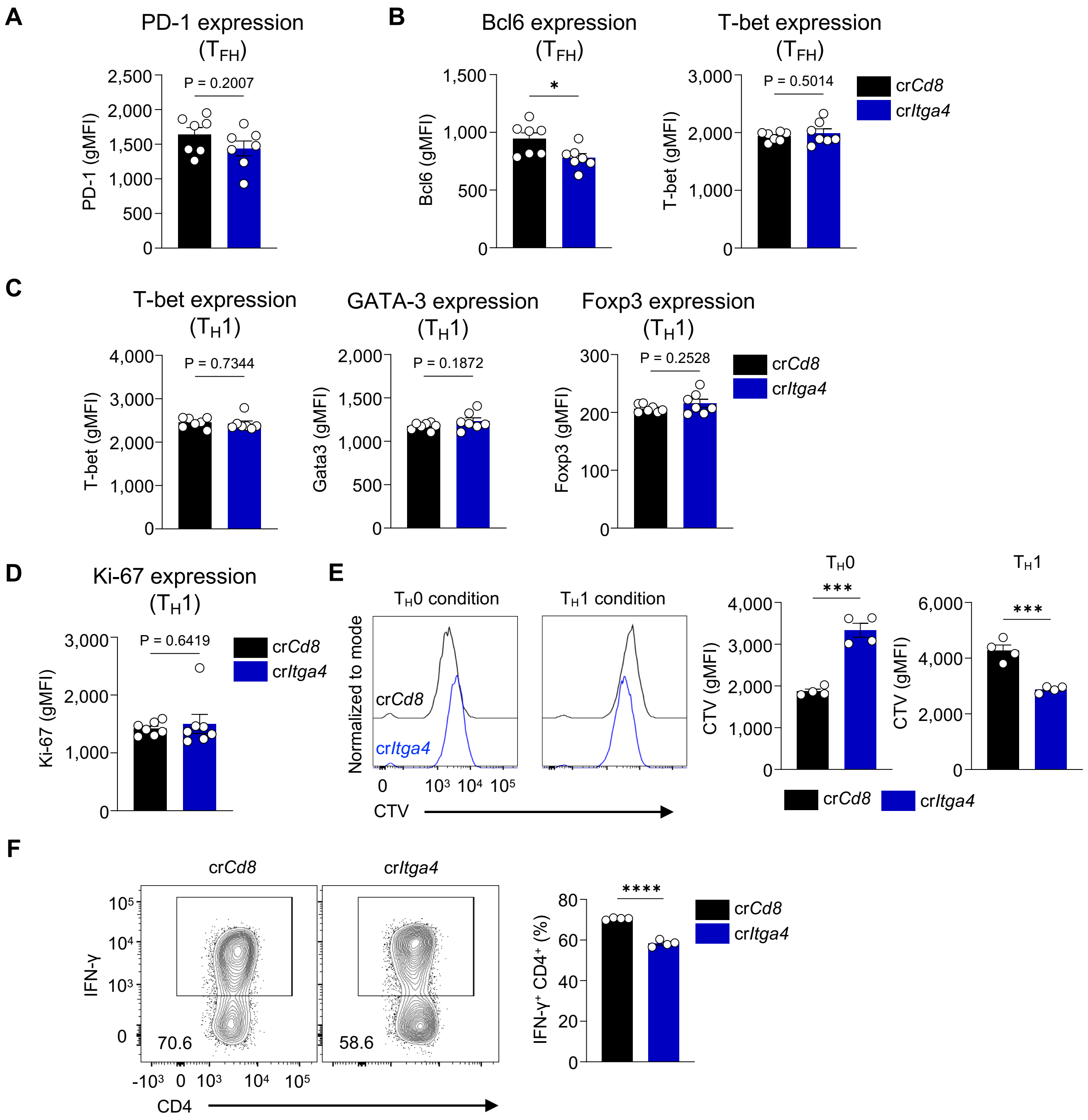

Supplementary Figure 4. Effect of *Itga4* disruption in CD4 T cells on T_H1 cell accumulation in the spleen during acute viral infection and *in vitro* T_H1 differentiation (A,B) During acute viral infection, the expression levels of PD-1, and transcription factors Bcl6, and T-bet were analyzed in T_{FH} cells (CXCR5$^+$ CD4$^+$) using flow cytometry. (C,D) During acute viral infection, the expression levels of transcription factors T-bet, GATA-3, Foxp3, and Ki-67 were analyzed in T_H1 cells (CXCR5$^-$ CD4$^+$) using flow cytometry. Each dot represents one mouse (n=7). (E,F) CD4 T cells cultured under T_H0 and T_H1 conditions were stained with CellTrace Violet (CTV) and analyzed by flow cytometry (n=4). Results are presented as mean ± SEM and were analyzed by using unpaired two-tailed Student's t-test. *$p < 0.05$; ***$p < 0.001$; ****$p < 0.0001$.
